# A Database of Solution Additives Promoting Mg^2+^ Dehydration and the Onset of MgCO_3_ Nucleation

**DOI:** 10.1021/acs.cgd.1c01525

**Published:** 2022-04-05

**Authors:** Dimitrios Toroz, Fu Song, Amira Uddin, Gregory A. Chass, Devis Di Tommaso

**Affiliations:** † Department of Chemistry, Queen Mary University of London, Mile End Road, London, E1 4NS, United Kingdom; ‡ Department of Chemistry and Chemical Biology, McMaster University, Hamilton, Ontario L8S 4M1, Canada; § Faculty of Land and Food Systems, The University of British Columbia, Vancouver, British Columbia V6T 1Z4, Canada

## Abstract

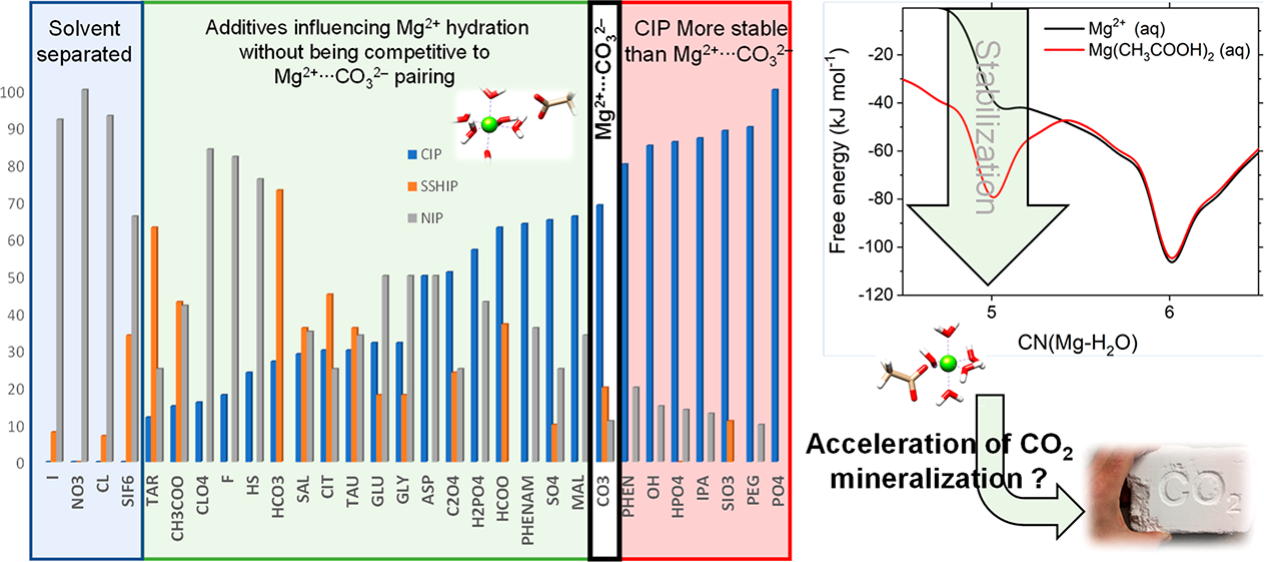

Formed via aqueous carbonation of Mg^2+^ ions, the crystallization of magnesite (MgCO_3_) is a promising route to carbon capture and reuse, albeit limited by the slow precipitation of MgCO_3_. Although magnesite is naturally abundant, forming at low temperature conditions, its industrial production is an energy-intensive process due to the temperatures required to prevent the formation of hydrated phases. The principal difficulty in aqueous conditions arises from the very strong Mg^2+^···H_2_O interaction, with high barriers to Mg^2+^ dehydration. Using atomistic simulations, we have investigated the influence of 30 additive anions (X^*n*–^, *n* = 1–3), ranging from simple halides to more complex molecules, on the first two steps of MgCO_3_ aggregation from solution, as follows: Mg^2+^ dehydration and subsequent prenucleative Mg^2+^···CO_3_^2–^ pairing. We have computed the thermodynamic stabilities of solvent shared ion pairs (Mg^2+^···H_2_O···X^*n*–^) and contact ion pairs (Mg^2+^···X^*n*–^) to reveal the propensity of solution additives to inhibit or promote Mg^2+^···CO_3_^2–^ formation. We have determined the stabilization of undercoordinated hydrated Mg^2+^ states with a vacant coordination site to which CO_3_^2–^ can bind, subsequently initiating MgCO_3_ nucleation or Mg^2+^ incorporation into the crystal lattice. Extensive molecular dynamics simulations of electrolyte solutions containing Na_2_CO_3_ with different sources of Mg^2+^ (i.e., MgCl_2_, MgSO_4_, and Mg(CH_3_COO)_2_) further show that the degree of dehydration of Mg^2+^ and the structure of prenucleation MgCO_3_ clusters change depending on the counterion identity. Through a fundamental understanding of the role of solution additives in the mechanism of Mg^2+^ dehydration, our results help to rationalize previously reported experimental observation of the effect of solvation environments on the growth of magnesite. This understanding may contribute to identifying the solution composition and conditions that could promote the low-temperature CO_2_ conversion into MgCO_3_ at industrially relevant scales.

## Abstract 2

We investigated the influence of solution additive anions on the early stages of MgCO_3_ nucleation. We computed the Mg^2+^ and anions pairing to reveal the propensity to inhibit or promote Mg^2+^···CO_3_^2−^ formation. We determined the stabilization of undercoordinated hydrated Mg^2+^ states to which CO_3_^2−^ can bind. We simulated the aggregation of MgCO_3_ clusters in the presence of additives.

## 1 Introduction

The mineralization of carbon dioxide (CO_2_) has the benefits of unlimited raw material supplement and longer-term storage carbonate materials, the output values of which are expected to reach $1 trillion per year by 2030.^1^ Therein, the anhydrous form of magnesium carbonate, magnesite (MgCO_3_), is widely used in food and fertilizers, in the manufacture of refractory materials, as a valuable construction material due to its fire-retardant properties, and in the production of eco-cements.^2^ Mining of MgCO_3_ exceeds 25 Mt year^–1^ with deposits concentrated in Russia, China, and Korea;^3^ hence worldwide usages are accompanied by transport costs. Conversely, magnesium ion (Mg^2+^) sources are globally widespread and plenty (accessible Mg silicate deposits estimated at 100 000 Gt),^4^ with MgCO_3_ able to be locally produced worldwide via mineral carbonation of Mg silicate.^5^ However, such CO_2_ mineralization into MgCO_3_ is limited by the slow rates of magnesite precipitation from solution.^5^ Its production is an energy-intensive process due to the high temperatures (*T* = 120–600 °C) required to prevent the formation of hydrated Mg carbonate phases such as nesquehonite (MgCO_3_·3H_2_O) and hydromagnesite (Mg_5_(CO_3_)_4_(OH)_2_·4H_2_O).^6^ Albeit of commercial use, these phases dominate industrial outputs and perpetuate the absence of local production of magnesite. The high temperature necessary to promote the direct precipitation of anhydrous MgCO_3_, the increased solid mass and volume of nesquehonite and hydromagnesite generated per mole of CO_2_ sequestered, as well as their inferior mechanical and structural properties, negatively impact on the cost, profitability, and thus industrial viability of Mg-mediated CO_2_ mineralization.^7^ The culprit slow precipitation rate of MgCO_3_ has long been ascribed to the very strong Mg^2+^···H_2_O interaction (hydration free energy of Mg^2+^ is −439 kcal mol^–1^),^8^ which raises the barrier of Mg^2+^ dehydration.^9^

Encouragingly, the solvation environment in which the mineral crystallization occurs may influence the Mg^2+^ dehydration process. In this regard, McKenzie et al. proposed that bisulfide delivered by sulfate reducing bacteria in sedimentary environments, although dilute, could catalyze the formation of natural dolomite (CaMg(CO_3_)_2_).^10^ Hence, to accelerate the synthesis of anhydrous MgCO_3_ under standard conditions, efforts have focused on the addition of salts,^11,12^ complexing compounds,^13^ alcohols,^14^ and microorganisms.^15^ However, there is a lack of understanding of additive identity and concentration, with few comprehensive studies resolving the effects of solution additives on the fundamental processes controlling the process of Mg^2+^ dehydration. We therefore looked to help bolster knowledge on the formation of Mg carbonates from aqueous solutions.

By providing a fundamental understanding of how the presence of solution additives can influence the rate-determining Mg^2+^ dehydration step, the composition of the solution may be rationally tuned to accelerate the kinetics of the early stages of MgCO_3_ nucleation and growth. In our recent study on the mechanism of Mg^2+^ dehydration, we have shown that Mg(H_2_O)_6_^2+^ is the only stable coordination state in pure water.^16^ However, solution additive anions such as fluoride, carboxylate, and bisulfide may stabilize undercoordinated configurations and subsequent incorporation into the lattice of magnesium carbonates, which could potentially promote low-temperature crystallization.^16^ Following these findings, herein we present a comprehensive computational investigation of the influence of thirty solution additives on the hydration properties of Mg^2+^ to determine which anions accelerate its dehydration as a function of the molecular size and functional groups of the additives. Table 1 reports the thirty solution additive ions (X^*n*–^, *n* = 1–3) considered in this study: ones that are naturally abundant in groundwater such as chloride (Cl^–^), fluoride (F^–^), sulfate (SO_4_^2–^), nitrate (NO_3_^–^), phosphates (H_*n*_PO_4_^3–*n*^, *n* = 0–2), silicate (SiO_3_^2–^), and (bi)carbonate (H)CO_3_^–^.^17^ Also ions that have been deemed important in promoting the formation of anhydrous forms of Mg carbonates include bisulfide (HS^–^) and carboxylic acids (HCOO^–^ and CH_3_COO^–^).^10,13^ Further, molecular ions containing multiple functional groups that may act cooperatively to promote Mg^2+^ dehydration such as taurate (C_2_H_6_NSO_3_^–^), aspartate (C_4_H_6_NO_4_^2–^), oxalate (C_2_O_4_^2–^), salicylate (C_7_H_5_O_3_^–^), citrate (C_6_H_5_O_7_^3–^), tartrate (C_4_H_4_O_6_^2–^), malate (C_4_H_4_O_5_^2–^), and aminophenolate (C_6_H_4_ONH_2_^–^) have been included. Peptides and alcohol molecules considered responsible for facilitating Mg^2+^ dehydration such as glycinate (C_2_H_4_NO_2_^–^), glutamate (C_5_H_8_NO_4_^–^), aspartate (C_4_H_6_NO_4_^2–^), and isopropyl alcohol ionic (C_3_H_7_O^2–^)^14,18−20^ were also considered. Finally, the hexafluorosilicate ion (SiF_6_^2–^) is produced on large scales in volcanoes^21^ and has been speculated to accelerate natural MgCO_3_ formation.^22^ Such a computational database may be used to identify conditions of solution compositions catalyzing the low-temperature CO_2_ conversion into MgCO_3_.

**Table 1:** Solution Additive Ions (X^*n*–^) Used to Assess the Effect of Solution Composition in Promoting Mg^2+^ Dehydration

X^*n*–^	formula	additive ion	abbreviation
1	Cl^–^	chloride	CL
2	F^–^	fluoride	F
3	I^–^	iodide	I
4	NO_3_^–^	nitrate	NO3
5	HCO_3_^–^	bicarbonate	HCO3
6	ClO_4_^–^	perchlorate	CLO4
7	CO_3_^2–^	carbonate	CO3
8	SO_4_^2–^	sulfate	SO4
9	HS^–^	bisulfide	HS
10	HCOO^–^	formate	HCOO
11	CH_3_COO^–^	acetate	CH3COO
12	PO_4_^3–^	phosphate	PO4
13	HPO_4_^2–^	hydrogen phosphate	HPO4
14	H_2_PO_4_^–^	dihydrogen phosphate	H2PO4
15	SiO_3_^2–^	metasilicate	SIO3
16	C_2_H_6_NSO_3_^–^	taurate	TAU
17	C_2_O_4_^2–^	oxalate	C2O4
18	C_7_H_5_O_3_^–^	salicylate	SAL
19	C_6_H_5_O_7_^3–^	citrate	CIT
20	C_4_H_6_NO_4_^2–^	aspartate	ASP
21	C_4_H_4_O_6_^2–^	tartrate	TAR
22	C_4_H_4_O_5_^2–^	malate	MAL
23	C_6_H_4_ONH_2_^–^	aminophenolate	PHENAM
24	C_2_H_4_NO_2_^–^	glycinate	GLY
25	C_5_H_8_NO_4_^–^	glutamate	GLU
26	OH^–^	hydroxyl	OH
27	C_6_H_5_O^–^	phenolate	PHEN
28	C_3_H_7_O^2–^	isopropyl alcohol ionic	IPA
29	C_8_O_5_H_16_^2–^	polyethylene glycol	PEG
30	SiF_6_^2–^	hexafluorosilicate	SIF6

We have used a combination of classical molecular dynamics (MD) and enhanced sampling metadynamics (MetaD) to characterize the ability of the solution additive ions (Table 1) to promote Mg^2+^ dehydration based on the following two well-defined molecular level criteria: formation of (1) solvent-shared ion pairs or (2) contact ion pairs with Mg^2+^, with either being less stable than Mg^2+^···CO_3_^2–^ (i.e., so as not to retard formation of the latter). These pairs can effectively stabilize undercoordinated hydrated Mg^2+^ states with a vacant coordination site (i.e., five-coordinated Mg^2+^) to which CO_3_^2–^ can bind, initiating the MgCO_3_ nucleation and/or incorporation of Mg^2+^ into the growing crystal lattice. Subsequently, we have conducted unbiased classical MD simulations of MgCO_3_ aggregation in the presence of selected additives to monitor the effect of solution composition, the dynamics of formation, and the structure of prenucleation clusters.

## 2 Computational Details

Classical MD simulations were performed with the use of GROMACS version 2016.3.^23^ The leapfrog algorithm with a time step of 2 fs was used to integrate the equations of motion. Simulations were conducted in the canonical (constant *NVT*) and isothermal–isobaric (constant *NPT*) ensembles at the target temperature *T* = 300 K and pressure *P* = 1 bar. The velocity rescale thermostat^24^ and the isotropic Parrinello–Rahman barostat^25^ were used with 0.4 ps and 2.0 ps as the thermostat and barostat relaxation times, respectively. The electrostatic forces were calculated by means of the particle-mesh Edwald approach with a cutoff of 1.2 nm. A 1.2 nm cutoff was also used for the van der Waals (vdW) forces. The LINCS algorithm was used at each step to preserve the bond lengths. Periodic boundary conditions were applied throughout.

The free energy profiles were obtained with well-tempered metadynamics,^26^ by using GROMACS 2016.3 equipped with the PLUMED 2.4.1 plugin.^27^ The distance between Mg^2+^ and the center of mass of the additive was used as a collective variable to compute the formation of ion pairs. The following two collective variables (CVs) were used to study the Mg^2+^ dehydration process: the Mg^2+^–water distance and the Mg^2+^–water coordination number (CN). The latter was defined using the continuous differentiable function:
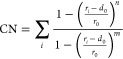
1where *r*_0_ = 1.1 Å, *d*_0_ = 1.9 Å, *n* = 4, and *m* = 8; *r*_*i*_ is the distance between Mg^2+^ and the oxygen atom of *i*th water molecule.^28^ The free energy profiles were constructed by running MetaD simulations with Gaussians laid every 1 ps and with an initial height equal to *k*_B_*T*. The Gaussian widths were 0.2 and 0.1 along the distance and coordination number (CN), respectively.^9,29^ The Supporting Information reports the input files of PLUMED, listing the parameters used to compute the free energy profiles as a function of coordination number and distance.

The solution additives were modeled by using the general AMBER force field (GAFF)^30^ to model the additives labeled NO_3_^–^, SIF_6_^–^, and HS^–^ and the AMBER-99^31^ force field to model the other molecular ions in Table 1. The Mg^2+^–water interactions were described by the Lennard–Jones GAFF potential together with the SPC/E water model,^32^ which we have previously shown to resolve structural, dynamic, and kinetic properties of hydrated Mg^2+^ in good agreement with quantum chemical and experimental data.^9^ Moreover, the use of the AMBER class of force field has allowed us to simulate the Mg^2+^ dehydration in the presence of other electrolytes using a consistent set of intra- and intermolecular force field parameters. The Antechamber package was used to compute the atomic partial charges in the framework of the restrained electrostatic potential formalism^33^ on the optimized structures and electrostatic potentials of the molecular ions determined with the Gaussian09 electronic structure code at the HF/6-31G(d) level of theory.^34^

The following protocol was used to generate the Mg^2+^ containing electrolyte solutions. We first conducted an MD (*NPT*) simulation of around 1400 water molecules for 1 ns to generate an equilibrated aqueous solution. This was used to generate Mg^2+^/X^*n*–^ solutions by randomly replacing two water molecules with one magnesium ion and one counterion. We then conducted a series of *NVT* simulations for Mg^2+^···X^*n*–^ separation distances (*d*) varying from 1.3 to 0.45 nm using a harmonic bias potential with a force constant of 500 kJ mol^–1^. Starting from the last configuration corresponding to a Mg^2+^···X^*n*^ distance of approximately 0.45 nm, we conducted MetaD simulations in the *NVT* ensemble for 100 ns, which is sufficient to obtain convergent free energy profiles as a function of the Mg^2+^–water coordination number as shown in Figure S1. For all additives assessed, the free energy profiles are the average of three different repeats to ensure statistical certainty. To evaluate the magnitude of the ability of each additive to promote Mg^2+^ dehydration, we have conducted two further sets of MetaD simulations with respect to Mg^2+^–water coordination as follows: in the first set the Mg^2+^···X^*n*–^ separation was kept at 0.45 nm, which corresponds to the position of the second Mg^2+^ hydration shell, by imposing a harmonic bias potential with a force constant of 1000 kJ mol^–1^ along the reaction coordinate defined as the distance between the two ions; in the second set, Mg^2+^ and X^*n*–^ were in direct contact.

## 3 Results

By influencing the hydration structure of Mg^2+^, inorganic ions and organic ligands in aqueous environments may activate relevant Mg^2+^ dehydration.^35^ In solution, interacting Mg^2+^ and X^*n*–^ could be in direct contact or bridged by water molecules. These states are labeled, respectively, as contact ion pair (CIP) and solvent-separated ion pair (SSIP) states.^36^ Ion pairs with a single water molecule spanning the ions are also sometimes called solvent-shared ion pairs (SSHIPs).^37^ The tendency of the magnesium and additive ions to form contact or solvent-separated pairs depends on the competition between Mg^2+^···H_2_O and Mg^2+^···X^*n*–^ interactions.

**Figure 1:**
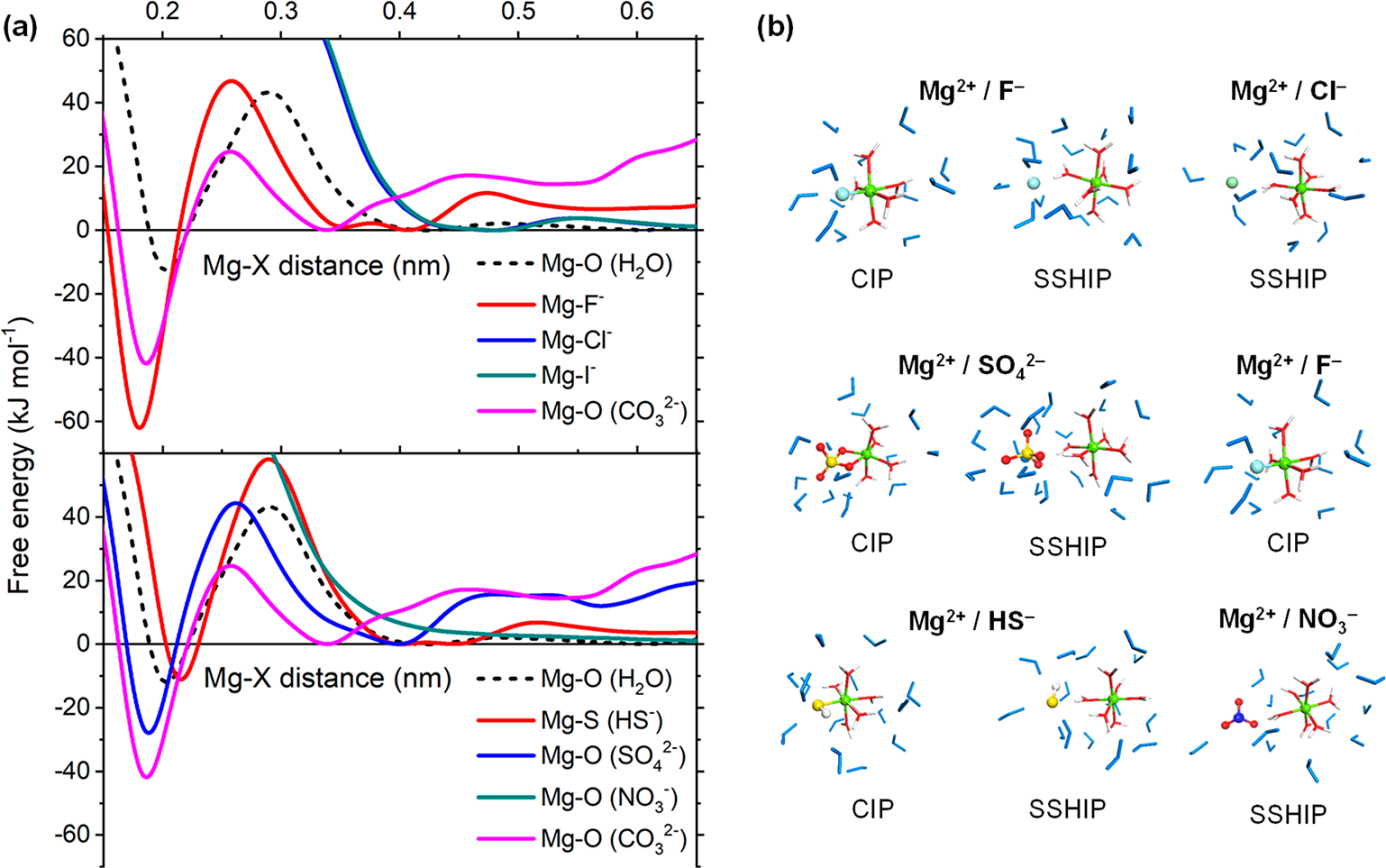
(a) Free energy as a function of the distance between Mg^2+^ and the center of mass of selected solution additive ions (X^*n*–^ = F^–^, Cl^–^, I^–^, HS^–^, SO_4_^2–^, NO_3_^–^). The profiles are compared with the free energy for the removal of a single water molecule from the first hydration shell of Mg(H_2_O)_6_^2+^. (b) Structures of selected contact ion pairs (CIPs) and solvent-shared ion pairs (SSHIPs) corresponding to the minima on the free energy profiles.

We have quantified the strength of ion pairing in terms of the free energy as a function of the Mg^2+^···X^*n*–^ distance (Figure 1a). We have reported the error bars on these free energy profiles in Figures S2 and S3. In the initial configuration, the Mg^2+^ and the counterion were separated by at least 0.4 nm and MetaD was then employed to compute the free energy profiles over separation distances up to 0.8 nm to determine which Mg^2+^/X^*n*–^ pairs form a thermodynamically stable contact ion. Analysis of the time series of the CV defined by the distance between Mg^2+^ and the center of mass of the solution additive ion (Figure S4) shows that both bind (CIP) and unbind (SSHIP) states are sampled during each repeat of the simulations used to produce the free energy profiles. The exception is PO4. Despite several attempts to capture the SSHIP state, using different starting points for the Mg^2+^ and PO_4_^2–^ ions, we have observed a strong binding with the formation of only the CIP state.

We have summarized the key features of the free energy profiles as a function of the Mg^2+^···X^*n*–^ distance in Table 2. For each Mg–X pair, the free energy of formation of the CIP (Δ*G*) was determined from the difference between the values in the free energy profile at the positions corresponding to the CIP, *r*_1_^min^, and SSHIP, *r*_2_^min^ (at ∼0.45 nm). Similarly, the standard Gibbs energy of activation (Δ^‡^*G*) was determined as the difference between the values at *r*_1_^min^ and the position of the transition state between CIP and SSHIP, *r*^max^. The free energy for the removal of a water molecule from the first hydration shell of Mg^2+^ has also been computed to determine if a particular Mg^2+^···X^*n*–^ contact ion pair is thermodynamically more stable than the hexahydrated complex [Mg(H_2_O)_6_]^2+^. For example, the free energy for the Mg^2+^···CO_3_^2–^ pairing (Δ*G* = −26 kJ mol^–1^) is significantly lower than that for [Mg(H_2_O)_6_]^2+^ (−7 kJ mol^–1^), while the Gibbs energy of activation of these CIPs is lower than that for Mg^2+^···H_2_O dissociation (Δ^‡^*G* = +48 kJ mol^–1^). Consequently, the Mg^2+^···CO_3_^2–^ CIP should be thermodynamically and kinetically favored with respect to [Mg(H_2_O)_6_]^2+^.

**Table 2:** Positions and Free Energies of Formation of Contact (CIP) and Solvent-Shared (SSHIP) Mg^2+^/X^*n*+^ Ion Pairs Computed from MetaD Simulations as a Function of Mg^2+^···X^*n*+^ Internuclear Distance^a^

additive	*r*_1_^min^	*r*^max^	*r*_2_^min^	Δ*G*	Δ^‡^*G*
PO4	0.176	–	–	–111.0	–
HCO3	0.191	0.394	0.254	–4.2	55.2
HCOO	0.191	0.387	0.254	–1.7	42.7
NO3	–	–	–	–	–
SIO3	0.189	0.351	0.266	–47.8	54.6
CO3	0.188	0.336	0.259	–42.7	25.0
HPO4	0.187	0.399	0.272	–54.4	40.0
PEG	0.187	0.370	0.264	–52.3	48.8
IPA	0.187	0.363	0.271	–49.2	49.9
OH	0.183	0.367	0.261	–51.9	52.1
PHEN	0.190	0.430	0.275	–39.8	45.3
SO4	0.190	0.401	0.260	–29.0	44.7
MAL	0.190	0.372	0.253	–16.7	38.5
C2O4	0.190	0.380	0.253	–23.5	30.8
H2PO4	0.193	0.411	0.270	–31.6	52.7
PHENAM	0.190	0.416	0.275	–41.2	47.5
SIF6	0.192	0.417	0.255	–0.6	20.1
CIT	0.189	0.407	0.266	–43.6	41.2
ASP	0.190	0.410	0.267	–34.3	51.9
TAU	0.193	0.411	0.277	–18.6	47.7
GLU	0.191	0.388	0.261	–23.6	47.4
GLY	0.190	0.374	0.254	–18.5	36.6
SAL	0.191	0.367	0.255	–16.9	37.6
TAR	0.190	0.373	0.253	–11.0	44.5
CH3COO	0.190	0.380	0.253	–15.7	42.0
HS	0.215	0.398	0.292	–12.1	58.4
F	0.184	0.409	0.261	–62.8	47.0
CLO4	0.202	0.428	0.287	–7.9	36.1
Cl	–	0.475	–	–	–
I	–	0.481	–	–	–
H2O	0.200	0.426	0.292	–11.9	43.5

a The values of *r*_1_^min^ and *r*_2_^min^ refer to the positions of the CIP and SSHIP on the free energy profile, and the value of *r*^max^ refers to the position of the transition state between CIP and SSHIP. The Gibbs free energies of reaction (Δ*G*) and standard Gibbs energy of activation (Δ^‡^*G*) are with respect to SSHIPs. The values are compared with those obtained for the removal of a single water molecule from hydrated Mg^2+^. Distances in nm and free energies in kJ mol^–1^.

The structures of the CIPs and SSHIPs of Mg^2+^ with selected counterions corresponding to the structures residing at a minimum on their respective free energy profiles are reported in Figure 1b. For example, the fluoride ion forms a very stable CIP with Mg^2+^ (Δ*G* = −63 kJ mol^–1^). The activation barrier for the formation of Mg^2+^···F^–^ (Δ^‡^*G* = 47 kJ mol^–1^) is higher than the free energy necessary to remove a water molecule from [Mg(H_2_O)_6_]^2+^ (Δ^‡^*G* = 44 kJ mol^–1^). For Cl^–^, I^–^, and NO_3_^2–^ the absence of a free energy minimum on the free energy profile corresponds to the absence of a contact ion pair. Also, NO_3_^2–^ does not even form an SSHIP state with Mg^2+^. For these ions, no disturbance in the Mg^2+^ inner hydration shell is seen prior to the energetically costly replacement of a water molecule with one chlorine, iodide, or oxygen (nitrate). Therefore, Cl^–^, I^–^, and NO_3_^2–^ have the tendency to form solvent-separated pairs with the magnesium ion. Our results confirm recent broadband dielectric relaxation spectroscopy measurements of aqueous MgCl_2_ solutions, which show no evidence for the significant formation of CIP.^38^ The dominant building unit in the magnesium sulfate solution, Mg(η^2^-SO_4_)(H_2_O)_4_^2+^, is reported in Figure 1b: the sulfate coordinates Mg^2+^ in a bidentate mode and the hydration number is 4, a result which agrees with static density functional theory calculations of hydrated MgSO_4_ cluster.^39^ The free energy profiles with the sulfate ion show a pronounced energy minimum corresponding to the formation of Mg(η^2^-SO_4_)(H_2_O)_4_^2+^, which is thermodynamically more stable than the Mg^2+^···H_2_O···SO_4_^2–^ SSHIP and the hexahydrated magnesium complex (Table 1). The activation energy of the formation of Mg^2+^–SO_4_^2–^ (Δ^‡^*G* = +45 kJ mol^–1^) is higher than that of Mg^2+^···H_2_O dissociation (Table 1). The CIP with HS^–^ has a stability similar to that of [Mg(H_2_O)_6_]^2+^, but the activation barrier of Mg^2+^···HS^–^ formation is significantly higher than the free energy necessary for the removal of a water molecule.

**Figure 2:**
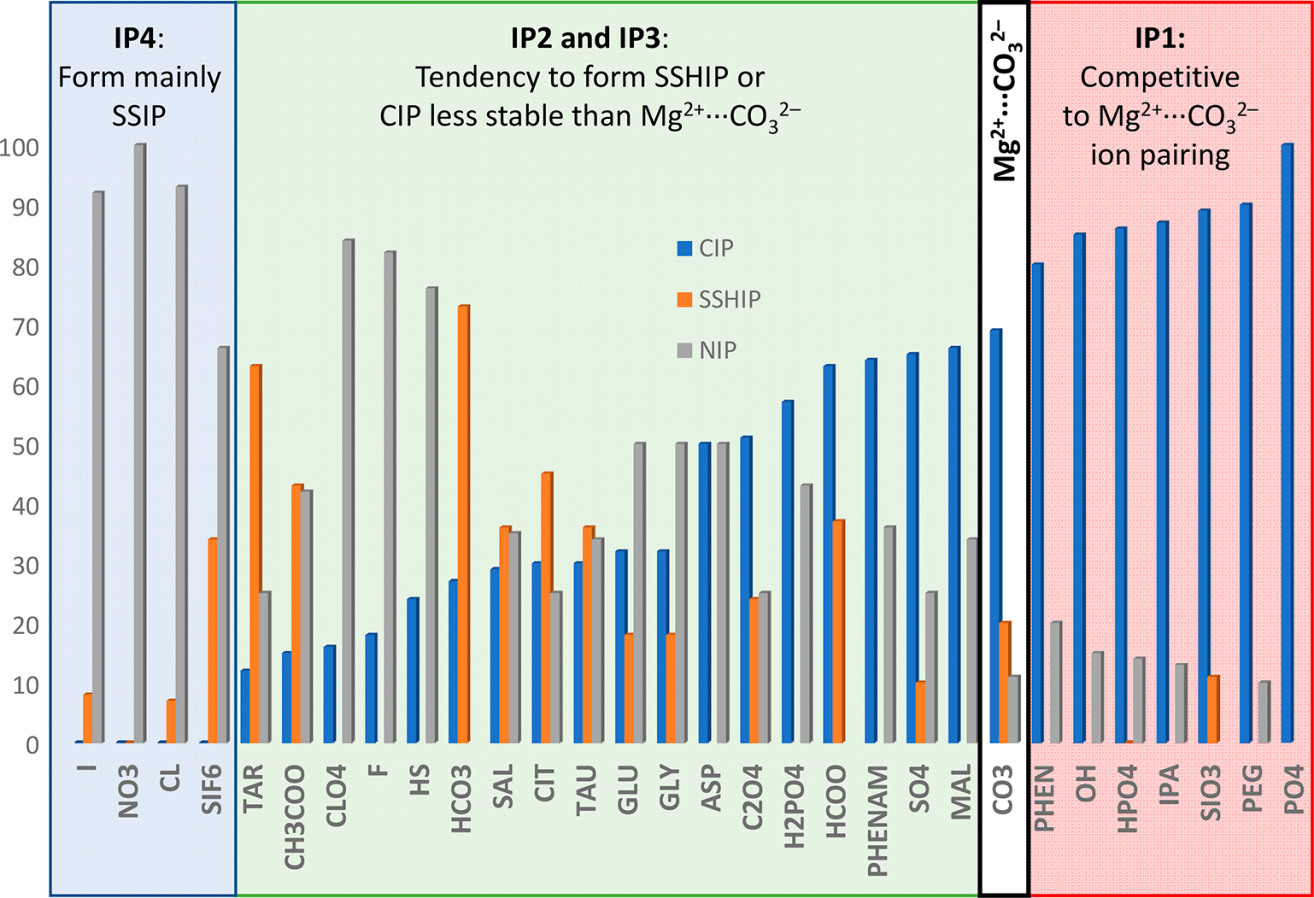
Distribution of contact ion pairs (CIPs), solvent-shared ion pairs (SSHIPs), and no-contact ion pairs (NIPs) between Mg^2+^ and X^*n*–^ obtained from the analysis of the MetaD simulations of Mg^2+^ containing electrolyte solutions.

Figure 2 reports the distribution of CIPs, SSHIPs, and SSIPs of Mg^2+^ with the additive anions obtained from the analysis of the MetaD simulations, where we have sorted the solution additives according to their energetic ease to form CIPs. Another important aspect to consider is the ability to compete with the formation of Mg^2+^···CO_3_^2–^, the building unit of magnesite. Based on the propensity to form CIPs, SSHIPs, or SSIPs and to inhibit/promote Mg^2+^···CO_3_^2–^ pairing, we have classified the additive anions into the following ion pairing (IP) categories: IP1, IP2, IP3, and IP4.

### IP1: PO4, PEG, SIO3, IPA, HPO4, OH, PHEN

These ions form CIPs that are thermodynamically more stable than [Mg(H_2_O)_6_]^2+^ and MgCO_3_ (higher distribution of CIPs compared to CO3). Since the ion pairing of Mg^2+^···X^*n*–^ is competitive with Mg^2+^···CO_3_^2–^, ions belonging to IP1 may inhibit the early stages of magnesite nucleation.

### IP2: MAL, SO4, PHENAM, HCOO, H2PO4, ASP, GLY, GLU, TAU, CIT, SAL, HCO3, F, ClO4, C2O4

These ions form stable CIPs compared to [Mg(H_2_O)_6_]^2+^ but without being competitive toward MgCO_3_ pairing (lower distribution of CIPs compared to CO3). These ions may promote Mg^2+^ dehydration without inhibiting the early stages of MgCO_3_ nucleation.

### IP3: HS, CH3COO

These ions form stable SSHIPs and tend to be in the second hydration shell of Mg^2+^. While not directly promoting Mg^2+^ dehydration through the formation of more stable CIPs than [Mg(H_2_O)_6_]^2+^, ions of type IP3 may perturb the hydrated Mg^2+^ coordination. Moreover, it is unlikely that HS and CH3COO will inhibit the early stages of MgCO_3_ nucleation.

### IP4: I, CL, NO3, SIF6

These ions are mainly located outside the second hydration shell of Mg^2+^. Consequently, they show no or little ability to form contact or solvent-shared ion pairs. An example is NO_3_. This ion forms only solvent-separated ion pairing and is unlikely to influence the Mg^2+^ dehydration process.

**Figure 3:**
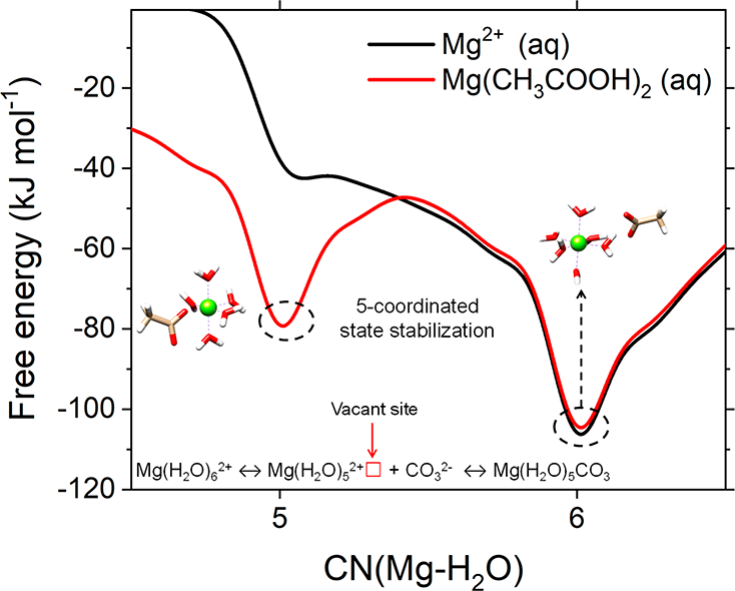
Free energy profiles of hydrated Mg^2+^ as a function of the ion–water coordination number obtained from MetaD simulations at *T* = 300 K.

The process of Mg^2+^ dehydration proceeds to a dissociative step^40^ and requires the formation of an undercoordinated pentahydrated intermediate [Mg(H_2_O)_5_^2+^].^16^ We have characterized the influence of counterions on the stabilization of undercoordinated Mg^2+^ states by computing the free energy profile as a function of the number of H_2_O molecules in the first hydration shell of the ion, which corresponds to the Mg^2+^–water coordination number (CN). The Gibbs free energy difference (Δ*G*_*i*→*j*_) and free energy barrier (Δ^‡^*G*_*i*→*j*_) between two coordination states *i* and *j* may give information on the transition between under- and over-coordinated states during the dynamics of Mg^2+^ (de)solvation.^41^ In Figure 3, results of MetaD simulations of hydrated Mg^2+^ show that in pure liquid water the sixfold coordination with water, Mg(H_2_O)_6_^2+^, is the most stable hydration state of Mg^2+^. The generation of a vacant site at the central magnesium ion corresponds to the transformation from the six- to the five-coordinated state to which carbonate can bind to initiate the MgCO_3_ nucleation or Mg^2+^ incorporation into the magnesite crystal lattice. However, the Mg(H_2_O)_6_^2+^ ↔ Mg(H_2_O)_5_^2+^ conversion is restricted by the high free energy barrier (Δ^‡^*G*_*i*→*j*_ ≈ 65 kJ mol^–1^). Conditions stabilizing the five-coordinated state will promote the Mg^2+^ dehydration process (Figure 3). Mergelsberg recently proposed that the greater salinity in natural systems may stabilize the five-coordinated intermediate.^42^ Similarly, the faster kinetics of MgCO_3_ precipitation measured within the nanoconfined water environments, compared to the bulk solution, was explained in terms of the reduction in coordinating water molecules (fewer than six) for Mg^2+^.^43^

To quantify the ability of each additive in Table 1 to promote the Mg^2+^ dehydration process, we have conducted MetaD simulations of electrolyte solutions where the separation between Mg^2+^ and X^*n*–^ was kept at approximately *d* = 0.45 nm by imposing a harmonic potential with a force constant of 1000 kJ mol^–1^ between magnesium and the counterion ion. This corresponds to the formation of SSHIP and allows us to evaluate the ability of solution additives to stabilize the undercoordinated Mg^2+^ states. Figure 3 shows that the presence of the acetate ion (CH_3_COO^–^) greatly stabilizes the five-coordinated Mg^2+^ state, promoting its dehydration. Power and co-workers proposed that the Mg^2+^ dehydration by surface-bound carboxyl groups promotes the low-temperature precipitation of dolomite on carboxylated polystyrene spheres.^44^ Therefore, our study demonstrates that at room temperature the presence of specific solution additives can stabilize undercoordinated complexes, promoting the subsequent steps of Mg carbonate nucleation and growth.

**Figure 4:**
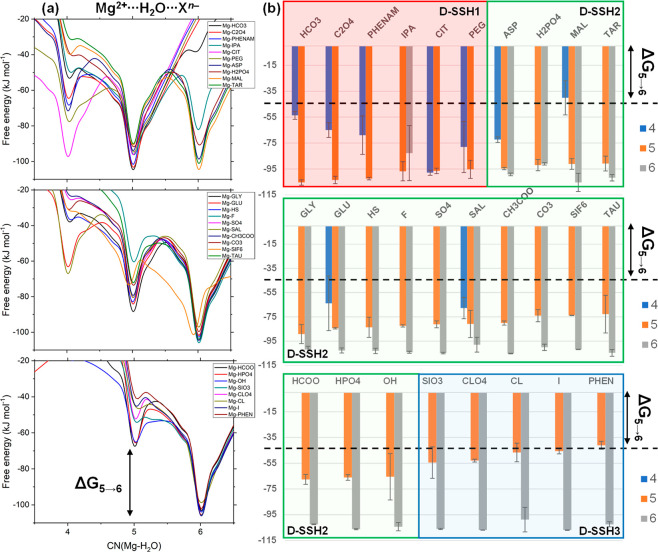
(a) Comparison of free energy profiles as a function of the Mg^2+^–H_2_O coordination number, CN(Mg–H_2_O), for solvated Mg^2+^ with a counterion in its second hydration shell (solvent-shared ion pairs, SSHIPs). (b) Free energies of [Mg(H_2_O)_4_]^2+^, [Mg(H_2_O)_5_]^2+^, and [Mg(H_2_O)_6_]^2+^ states of Mg^2+^ in solutions containing additive anions (X^*n*–^) with X^*n*–^ forming an SSHIP with Mg^2+^.

The ability of additives to replace water molecules when they form SSHIPs with Mg^2+^ may accelerate the nucleation events by increasing the proportion of undercoordinated Mg^2+^ species without being competitive with the desired MgCO_3_ ion pairing. The free energy profiles as a function of the Mg^2+^–H_2_O coordination number, CN(Mg–H_2_O), for solvated Mg^2+^ with a counterion in its second hydration shell (solvent-shared ion pairs, SSHIPs) are reported in Figure 4a, from which we have extracted the values of the free energies of the four, [Mg(H_2_O)_4_]^2+^; five, [Mg(H_2_O)_5_]^2+^; and six, [Mg(H_2_O)_6_]^2+^, coordination states of Mg^2+^ in solutions containing X^*n*–^ forming SSHIPs with Mg^2+^. The error bars on the free energy profiles as a function of the Mg^2+^–H_2_O coordination number are shown in Figure S5. We have identified the following subsets of additives based on the propensity of a counterion to stabilize undercoordinated (four and five) states with respect to Mg(H_2_O)_6_^2+^ (Figure 4b), which promotes dehydration even when they form solvent-shared ion pairs (D-SSH): D1-SSH, D2-SSH, and D3-SSH.

### D1-SSH: PEG, CIT, IPA, PHENAM, C2O4, HCO3

These ions highly stabilize the five-coordination state, which becomes thermodynamically preferred over the six-coordinate one [Mg(H_2_O)_6_^2+^]. We can also observe the appearance of a minimum on the free energy profile that corresponds to a tetrahydrated complex (Mg(H_2_O)_4_^2+^, Figure 4a). PEG and IPA are, however, highly competitive toward Mg^2+^···CO_3_^2–^ pairing (Figure 2). This class of ions could inhibit the early stages of aqueous magnesite formation.

### D2-SSH: SAL, GLU, GLY, TAR, MAL, H2PO4, ASP, OH, HPO4, HCOO, TAU, SIF6, CH3COO, SO4, F, HS

The presence of one of these ions in the second hydration shell of Mg^2+^ leads to a statistically significant stabilization (outside the error bars) of the five-coordination state compared with Mg^2+^ in pure liquid water. However, OH and HPO4 tend to form competitive CIPs with Mg^2+^···CO_3_^2–^ pairing, and SIF6 forms mainly solvent-separated ion pairs. Otherwise, all other ions can be considered as suitable to dehydrate magnesium.

### D3-SSH: SIO3, PHEN, I, CL, CLO4, NO3

In the presence of these ions, the free energy difference between the five- and six-coordination states, Δ*G*_5→6_, is close to that to that in pure water. These ions have, therefore, very little effect on the dehydration of Mg^2+^ and are unlikely to promote the early stages of MgCO_3_ aggregation.

A similar analysis conducted for the solvated Mg^2+^ with a counterion in its first hydration shell (Figures S6 and S7) shows the stabilization of states with only three and four water molecules coordinated to Mg^2+^. However, such a situation would lead to a reaction pathway where the formation of the building unit of magnesite would require the CO_3_^2–^ to exchange with the counterion to form the building unit of magnesite: Mg^2+^···X^*n*–^ → Mg^2+^···CO_3_^2–^ + X^*n*–^. For this transformation to be thermodynamically possible, the Mg^2+^···X^*n*–^ CIP must be less stable than the Mg^2+^···CO_3_^2–^ CIP, which occurs for additives belonging to the IP2, IP3, and IP4 groups, according to the ion pair distribution analysis. We have identified the following subsets of additives based on the propensity of a counterion in the first hydration shell of Mg^2+^ to stabilize undercoordinated (three- and four-coordinated) states (D-CIPs): D-CIP1, D-CIP2, D-CIP3, and D-CIP4.

### D-CIP1: PO4, HPO4, H2PO4, CO3

The most stable hydrated states of Mg^2+^ when coordinated with these ions have only three water molecules. However, PO4 and HPO4 form more stable CIPs with Mg^2+^ than the carbonate ion.

### D-CIP2: PHENAM, TAR, PEG, MAL, CIT, C2O4, and SO4

The most stable hydrated states of Mg^2+^ when coordinated with these ions have four water molecules. Moreover, these ions are less competitive than Mg^2+^···CO_3_^2–^.

### D-CIP3: HCO3, HS, IPA, OH, CH3COO, GLU, ASP, SAL, PHEN, HCOO, F, SIO3, SIF6, TAU, GLY

All these ions stabilize a coordination number of five. From these ions HS, HCO3, IPA, OH, and CH3COO showed higher propensities to stabilize the five-coordinated state. However, a subset of additives show competitive energy release on stabilizing the five- and six-coordination states (TAU, GLY, and SIF6) with energy differences within 5 kJ mol^–1^. This result implies that additives in D-CIP3 can form a nonstable contact ion pair, which can spontaneously detach from the Mg^2+^ species and show higher mobility when interacting with water molecules.

### D-CIP4: CLO4, CL, NO3, I

These ions have a distinct preference to only stabilize the six-hydration state, Mg(H_2_O)_6_^2+^, without having any ability for contact ion paring with Mg^2+^.

We have examined in Table 3 the additives considered in the present study to promote Mg^2+^ dehydration, without being competitive with the formation of the building unit of magnesite, Mg^2+^···CO_3_^2–^ CIP, based on the following three criteria.

**Table 3:** Summary of the Ability of Solution Additive Anions to Promote Mg^2+^ Dehydration Based on Criteria 1–3^a^

	criterion 1	criterion 2	criterion 3	promote?
CH3COO	√	√		
HS	√	√		
HCO3	√	√		
CIT	√	√	√	Y
PHENAM	√	√	√	Y
C2O4	√	√	√	Y
SO4	√	√	√	Y
MAL	√	√	√	Y
GLU	√	√		
GLY	√	√		
SAL	√	√		
H2PO4	√	√	√	Y
ASP	√	√		
HCOO	√	√		
TAU	√	√		
F	√	√		
PEG		√		
IPA		√		
HPO4		√	√	
OH		√		
SIO3		√		
PHEN		√		
SIF6		√		
CLO4		√		
CL				
I				
NO3				
PO4			√	
TAR			√	

a Criterion 1. Mg^2+^ interaction with X^*n*–^; competition with Mg^2+^···CO_3_^2–^ pairing: A solution additive should form Mg^2+^···H_2_O···X^*n*–^ SSHIP, or Mg^2+^···X^*n*–^ CIP should be less stable than Mg^2+^···CO_3_^2–^. Criterion 2. Stabilization of undercoordinated Mg^2+^ states; influence of counterions on the Mg^2+^ dehydration kinetics: Mg^2+^···H_2_O···X^*n*–^ SSHIP should stabilize undercoordinated Mg(H_2_O)_5_^2+^ compared with the hexaaquo Mg(H_2_O)_6_^2+^ complex. Criterion 3. Stabilization of low hydration Mg(X)(H_2_O)_*m*_^2–*n*^ number states: For Mg^2+^···X^*n*–^ CIP the Mg(X)(H_2_O)_3_^2–*n*^ and Mg(X)(H_2_O)_4_^2–*n*^ complexes should be the most stable in solution.

Criterion 1 is the competition between X^*n*–^ and CO_3_^2–^ ion pairing with Mg^2+^: A solution additive should preferentially form Mg^2+^···H_2_O···X^*n*–^ SSHIPs or Mg^2+^···X^*n*–^ CIPs that are *less* stable than Mg^2+^···CO_3_^2–^.

Criterion 2 is the stabilization of undercoordinated Mg^2+^ states: X^*n*–^ in the second coordination shell of Mg^2+^, Mg^2+^···H_2_O···X^*n*–^ SSHIP, should stabilize undercoordinated Mg(H_2_O)_5_^2+^ compared with the hexaaquo Mg(H_2_O)_6_^2+^ complex.

Criterion 3 is the stabilization of low hydration Mg(X)(H_2_O)_*m*_^2–*n*^ states: X^*n*–^ directly coordinated to Mg^2+^, Mg^2+^···X^*n*–^ CIP, should stabilize Mg(X)(H_2_O)_3_^2–*n*^ and Mg(X)(H_2_O)_4_^2–*n*^ complexes.

The reported analysis provides a fundamental understanding of the role of solution additives in the Mg^2+^ dehydration process and could help rationalize experimental observation of the effect of solvation environments on the growth of Mg carbonates. A more detailed analysis based on the IP, D-SSH, and S-CIP classification has also been reported in Table S2.

**Figure 5:**
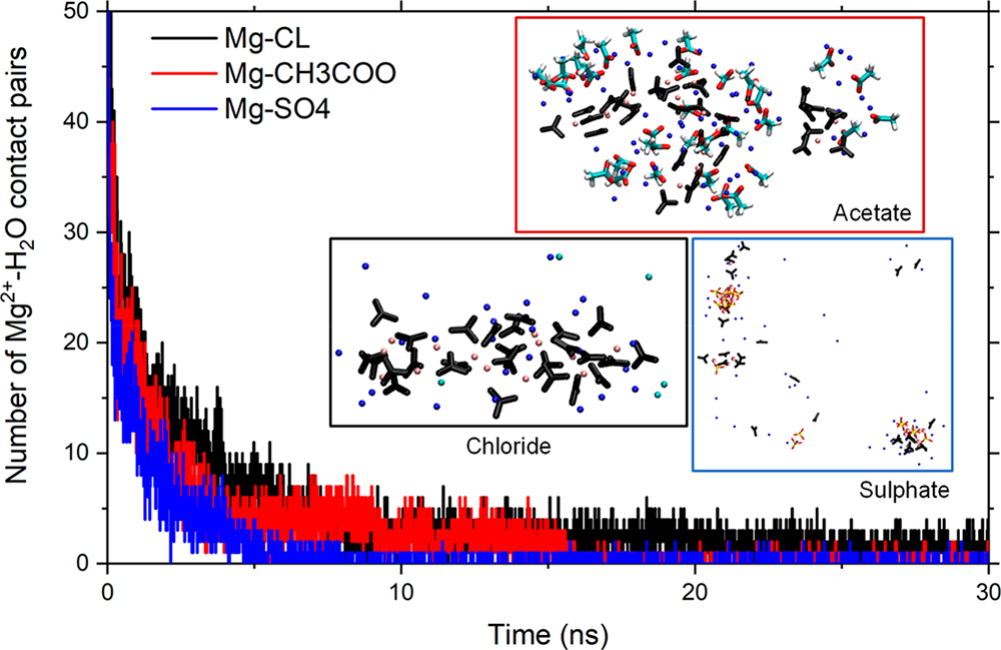
Progressive contact pairs of Mg^2+^ with oxygen atoms of water molecules. Snapshots of MgCO_3_ clusters forming in the presence of acetate, chloride, and sulfate ions.

We have further investigated the effects of selected additives on the formation of prenucleation MgCO_3_ clusters by conducting MD simulations (>50 ns) of three aqueous electrolyte solutions containing 1 mol dm^–3^ Na_2_CO_3_ and 0.5 mol dm^–3^ MgCl_2_, MgSO_4_, and Mg(CH_3_COO)_2_, respectively. These solutions were generated by ensuring that each Mg^2+^ ion in the first configuration of the simulation was fully hydrated (i.e., started out as Mg(H_2_O)_6_^2+^). Figure 5 shows the number of Mg^2+^···H_2_O pairs as a function of the simulation time, which decreases rapidly indicating that within the first few nanoseconds there is complete dehydration of Mg^2+^ and formation of the first MgCO_3_ clusters, an initiation of crystallization that would be very difficult to observe with experimental techniques. The tendency of dehydration and consequent MgCO_3_ aggregation follows the trend SO_4_^2–^ > CH_3_COO^–^ > Cl^–^ and agrees with what was observed from metadynamics calculations of the Mg^2+^ dehydration process.

## 4 Conclusions

The precipitation of anhydrous MgCO_3_, a route for the storage and functional utilization of carbon dioxide, is a slow process which has been linked to the very strong Mg^2+^···H_2_O interaction, which raises the barrier of Mg^2+^ dehydration. Solution environments could be highly influential to the molecular processes controlling the kinetics of the early stages of magnesite formation from solution. The difficulty of experimentally tracking the early stages of MgCO_3_ nucleation can be complemented by computational insights into the structural and energetic contributions of the nucleation sites and solution additives. In this study, we have used a combination of atomistic simulations, based on molecular dynamics and enhanced sampling (metadynamics) techniques, to investigate the effect of 30 differing additive anions, ranging from simple halides to more complex molecules, on the first two stages of MgCO_3_ nucleation, as follows: Mg^2+^ dehydration and subsequent Mg^2+^···CO_3_^2–^ pairing. Based on the calculation of the thermodynamic stabilities of solvent-shared ion pairs (Mg^2+^···H_2_O···X^*n*–^) and contact ion pairs (Mg^2+^···X^*n*–^), and the stabilization of undercoordinated hydrated Mg^2+^ states, we have classified additives based on their ability to promote Mg^2+^ dehydration without inhibiting the formation of the Mg^2+^···CO_3_^2–^ contact ion pair, the building block of magnesite. Further simulations of the formation of MgCO_3_ clusters in the presence of chlorine, acetate, and sulfate ions show the effect of the additives on the aggregation process as well. The findings of our study may guide us to reveal the role of the solution in the early stages of mineral formation and inspire the design of novel experiments assessing the effect of the additives in our database on aqueous MgCO_3_ formation.

## Supporting Information Available

Input files of PLUMED to compute the energy profile as a function of Mg^2+^-water distance and coordination number (CN); free energy profiles of hydrated Mg^2+^ as a function of Mg^2+^–water CN; energy profiles of ion pairing between Mg^2+^ and solution additive anions; time series of collective variable in metadynamics simulations defined by distances between Mg^2+^ and bicarbonate, formate, and sulfate additive anions; counterion effect on stabilization of undercoordinated Mg^2+^ and classification of solution additive anions; classifications of solution additive ions (PDF)
