# Supplementary material for: A Database of Solution Additives Promoting Mg2+ Dehydration and the Onset of MgCO3 Nucleation
Source: Cryst Growth Des. 2022 Apr 5;22(5):3080–9. doi: 10.1021/acs.cgd.1c01525 (PMC9073943; doi:10.1021/acs.cgd.1c01525)
Supplement: Supplementary file 1 — cg1c01525_si_001.pdf [file cg1c01525_si_001.pdf]

Supporting Information for **"A database of Solution additives promoting Mg^{2+} dehydration and the onset of $MgCO_3$ nucleation"**

Dimitrios Toroz,[a] Fu Song,[a] Amira Uddin,[a] Gregory A. Chass,[a,b,c] and Devis Di Tommaso *[a]*

[a] Department of Chemistry, Queen Mary University of London, Mile End Road, London, E1 4NS, UK

[b] Department of Chemistry and Chemical Biology, McMaster University, Hamilton, Ontario, L8S 4M1, Canada

[c] Faculty of Land and Food Systems, The University of British Columbia, Vancouver, British Columbia, V6T 1Z4, Canada

Corresponding Authors

* E-mail: g.chass@qmul.ac.uk, d.ditommaso@qmul.ac.uk

1. Computational details

1.1 Input files of metadynamics simulations with PLUMED

Input file to compute the energy profile as a function of coordination number

```
# RESTART

D1: DISTANCE ATOMS=2644,2645
restraint-d2: RESTRAINT ARG=d2 KAPPA=1000 AT=0.50

COORDINATION ...
LABEL=coord
GROUPA=2644
GROUPB=1-2643:3
SWITCH={RATIONAL R_0=0.12 D_0=0.19 NN=4 MM=8 D_MAX=0.35}
NLIST
NL_CUTOFF=0.4
NL_STRIDE=10
... COORDINATION

METAD ...
LABEL=metad
ARG=coord
SIGMA=0.1
HEIGHT=1.0
BIASFACTOR=5
TEMP=300.0
PACE=1000
GRID_MIN=0.
GRID_MAX=14.
GRID_BIN=1400
REWEIGHTING_NGRID=1400
... METAD

PRINT ARG=coord, metad.bias, d1,restraint-d1.bias STRIDE=10 FILE=COLVAR

ENDPLUMED
```

Input file to compute the energy profile as a function of distance

```
# restart previous simulation
# RESTART

# compute distance between atoms 4127 (Mg) and 4 (O1)
d1: DISTANCE ATOMS=4322,14
```

```
METAD ...
LABEL=metad
ARG=d1
PACE=1000
HEIGHT=1.0
SIGMA=0.02
FILE=HILLS
BIASFACTOR=5.0
TEMP=300.0
... METAD

UPPER_WALLS ...
ARG=d1
AT=0.8
KAPPA=2000.0
EXP=2
EPS=1
OFFSET=0.
LABEL=uwall
... UPPER_WALLS

PRINT STRIDE=500 ARG=d1,metad.bias FILE=COLVAR

ENDPLUMED
```

1.1 Free energy profiles as a function of the Mg^{2+}-water coordination number

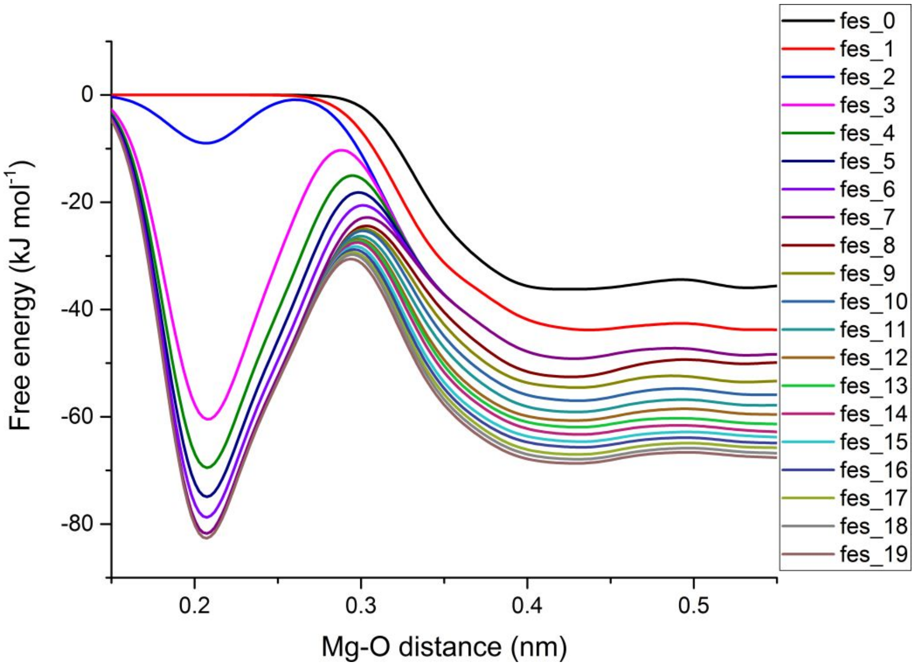

Figure S1. Evolution of the estimated free energy of the $Mg^{2+}\cdots H_2O$ showing that a convergent profile is obtained for a simulation period of 30 ns (fes_19).

2. Ion pairing between Mg^{2+} and solution additive anions

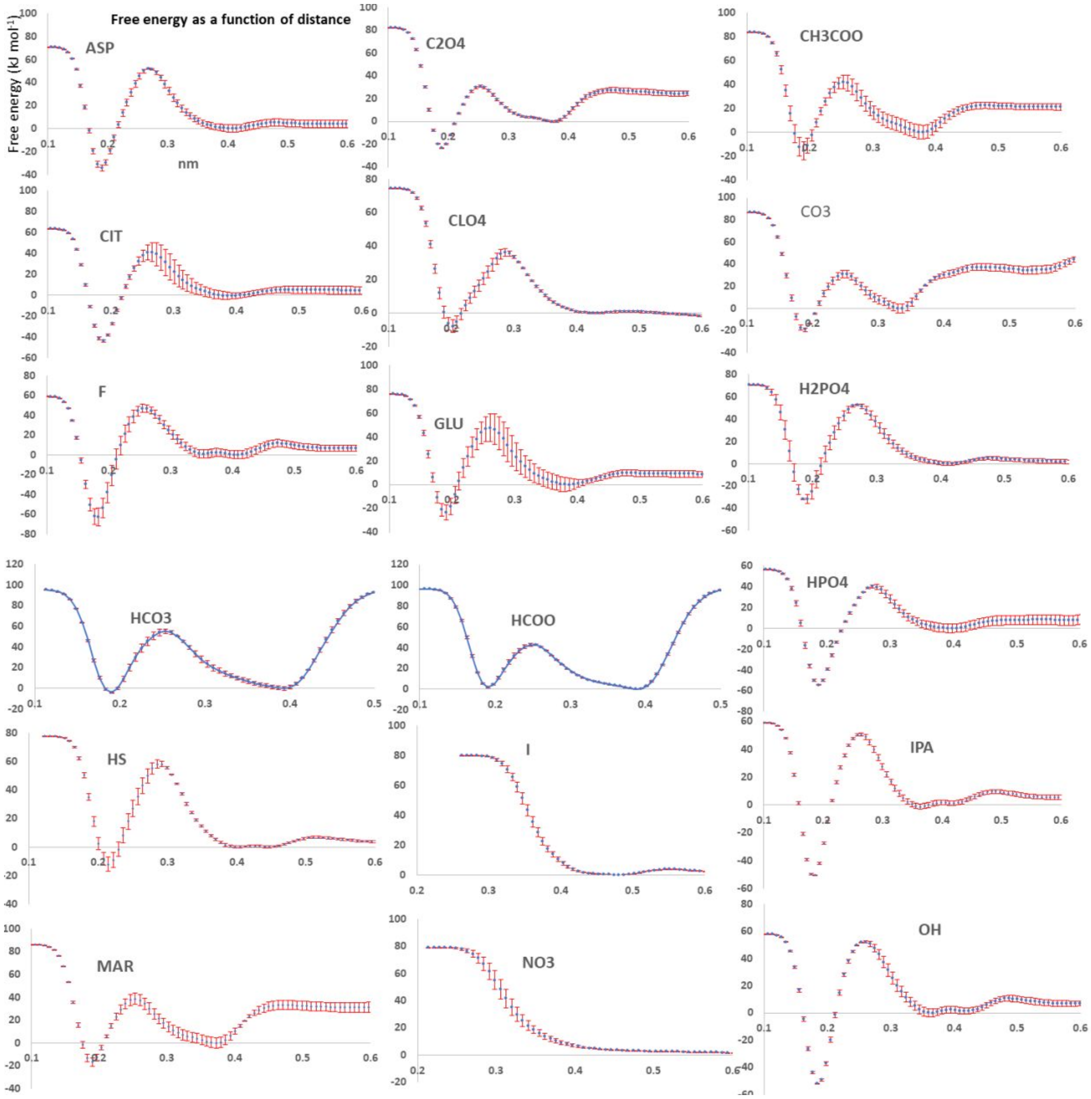

Figure S2. The free energy as a function of the distance (in nm) between Mg^{2+} and the center of mass of solution additive ions. Standard deviations were obtained from three repeats.

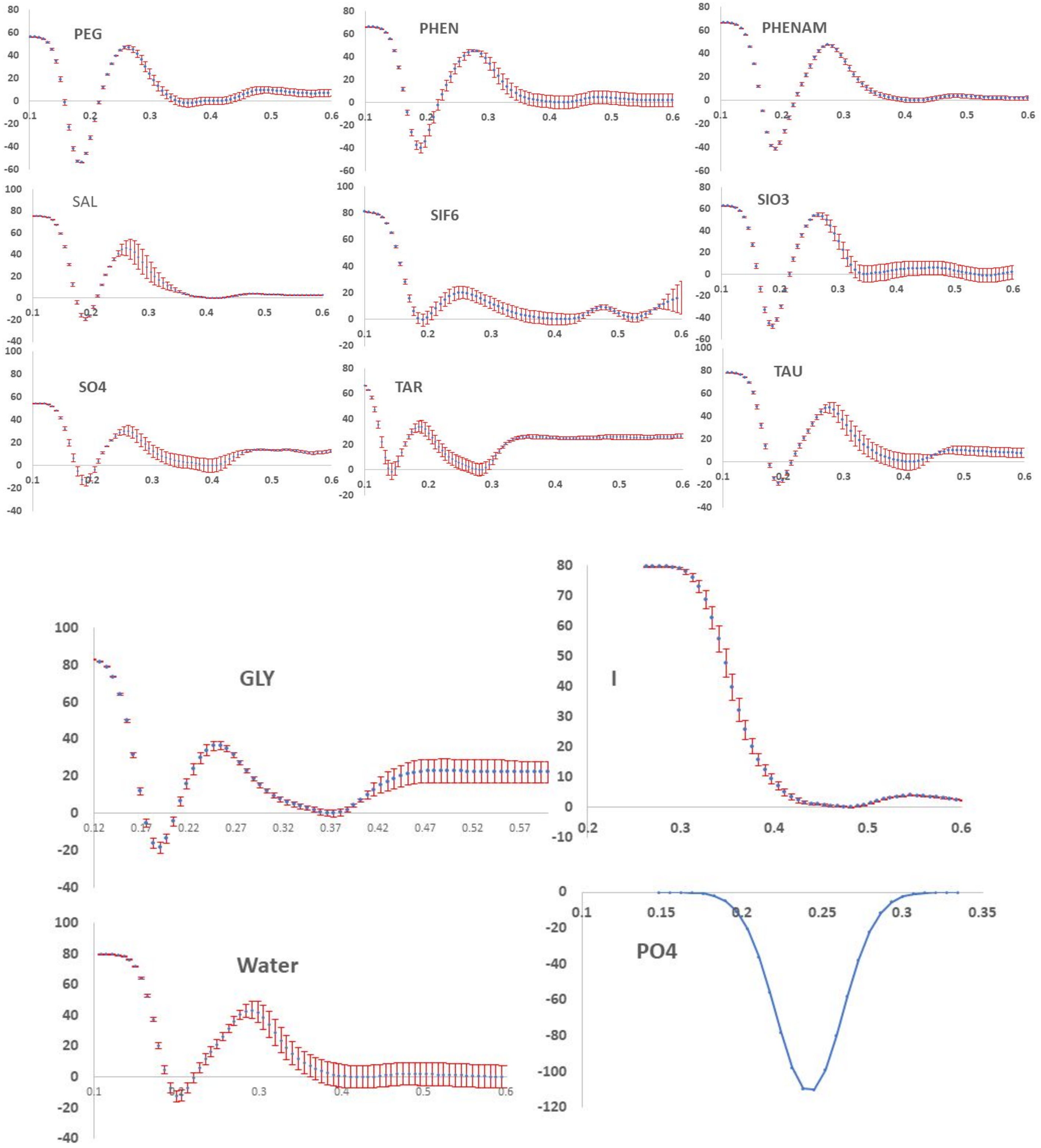

Figure S3. The free energy as a function of the distance (in nm) between Mg^{2+} and and the center of mass of solution additive ions. Standard deviations were obtained from three repeats.

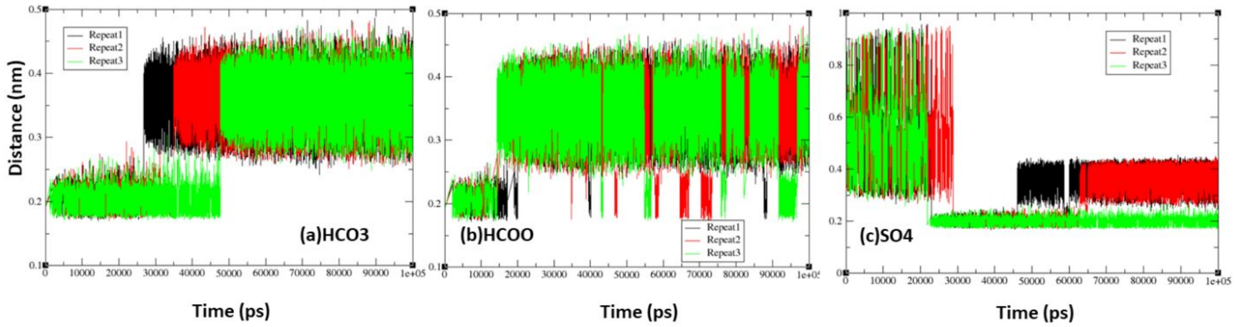

Figure S4. Time series of the collective variable in metadynamics simulations defined by the distance between Mg^{2+} and the centre of mass of the (a) bicarbonate (HCO3), (b) formate (HCOO), and (c) sulphate (SO4) additive anions.

3. Counterions effect on the stabilization of undercoordinated Mg^{2+}

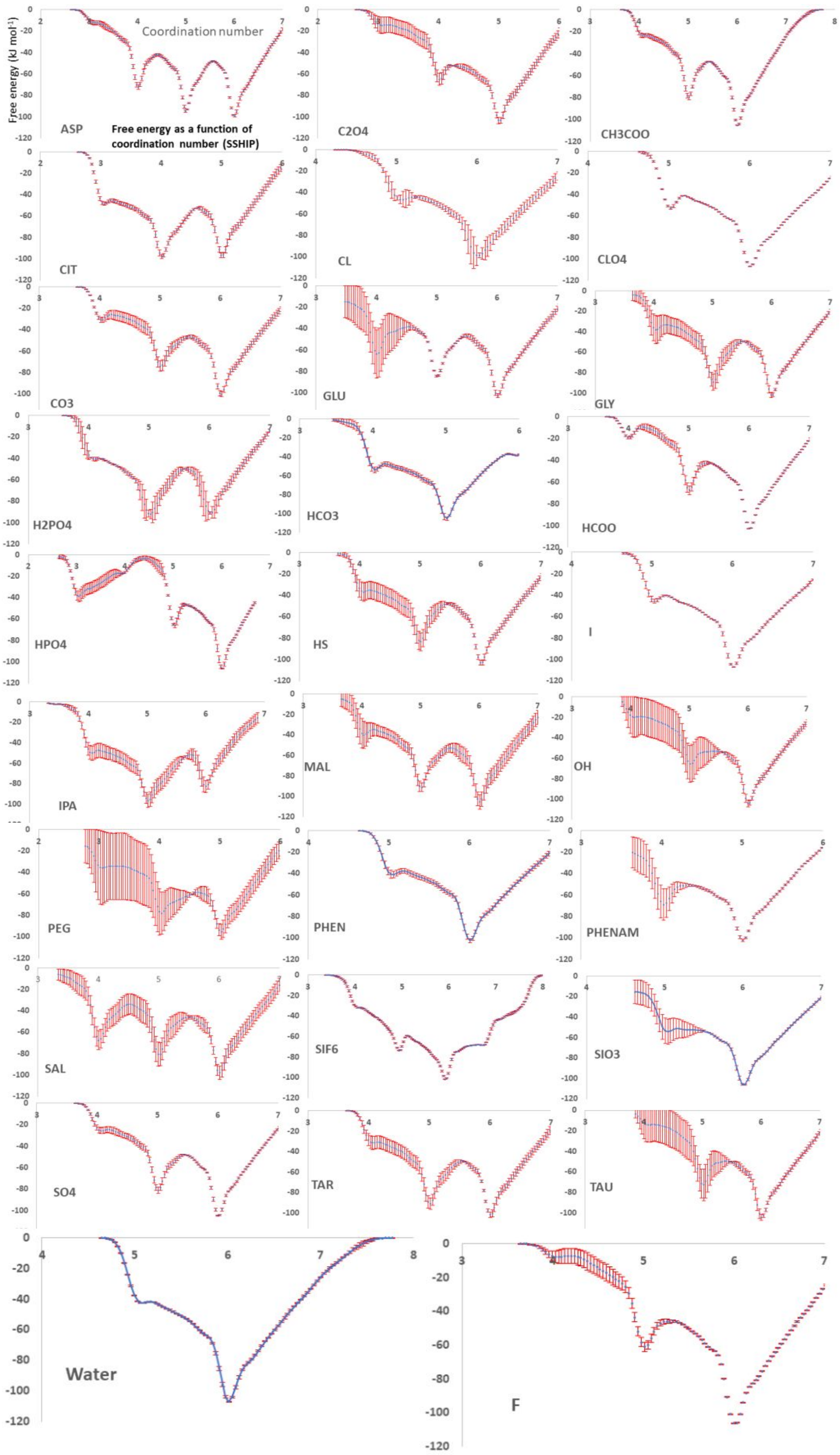

Figure S5. The free energy as a function of the $Mg^{2+}\cdots H_2O$ coordination number for solvated Mg^{2+} with a counterion in its second hydration shell (solvent-shared ion pair). Standard deviations were obtained from three repeats.

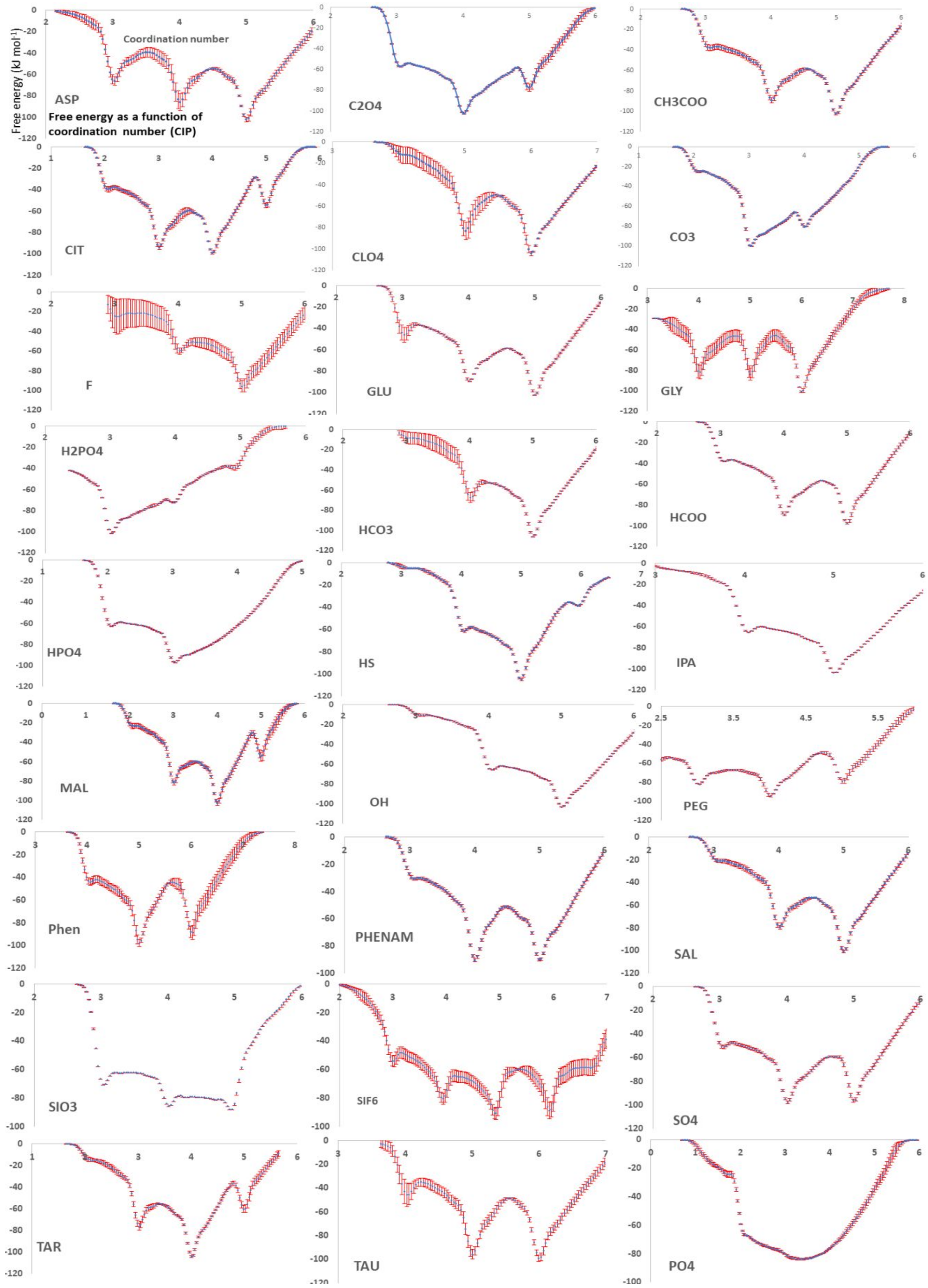

Figure S6. The free energy as a function of the $Mg^{2+}\cdots H_2O$ coordination number for solvated Mg^{2+} with a counterion in its first hydration shell (contact ion pair). Standard

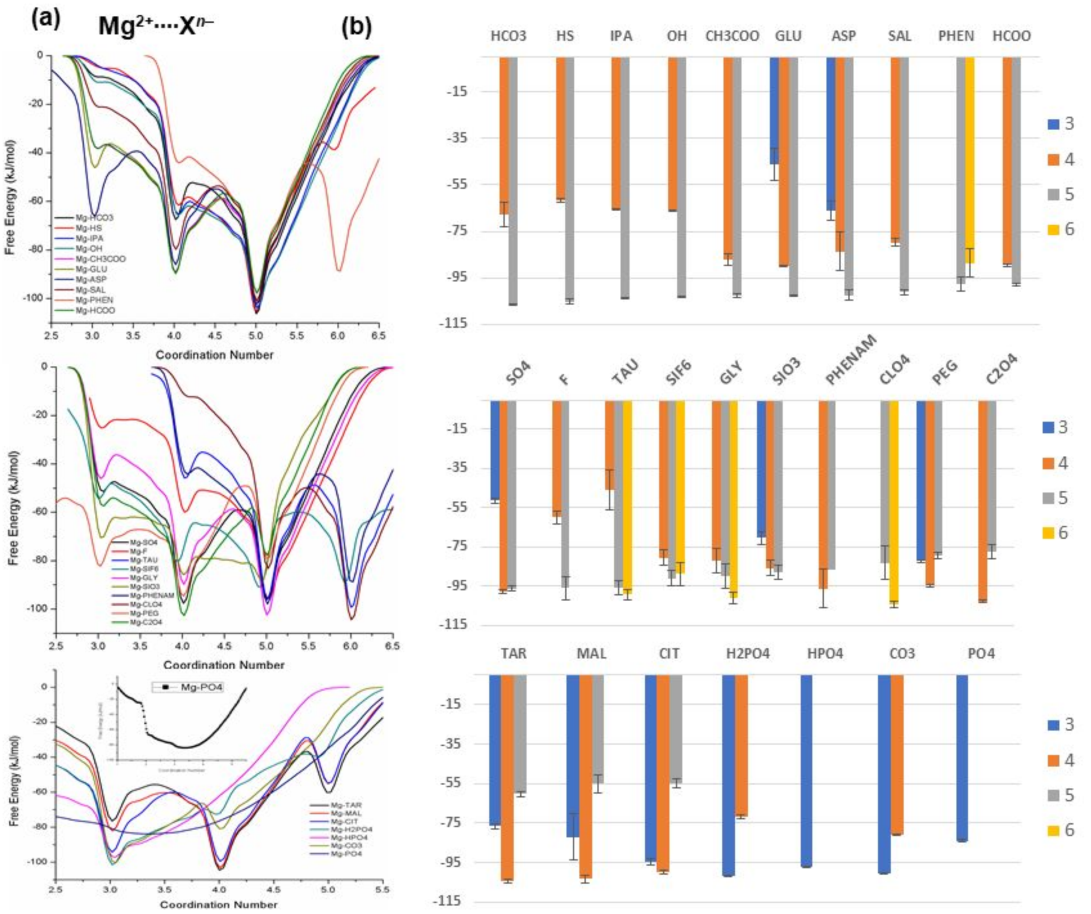

Figure S7. (a) Comparison of the free energy profiles as a function of the $Mg^{2+}\cdots H_2O$ coordination number, CN(Mg-H_2O), for hydrated Mg^{2+} (single Mg^{2+}, no counterions) and solvated Mg^{2+} with a counterion in its first hydration shell (contact ion pair, CIP). (b) Free energies of the $[Mg(H_2O)_3]^{2+}$, $[Mg(H_2O)_4]^{2+}$, $[Mg(H_2O)_5]^{2+}$, and $[Mg(H_2O)_6]^{2+}$ states of Mg^{2+} in solutions containing additive anions (X^{n-}) with X^{n-} forming a CIP with Mg^{2+}.

4. Classification of solution additive anions

Table S1. Classifications of the solution additive ions based on their behaviour towards $Mg^{2+}\cdots X^{n-}$ ion pairing and stabilization of undercoordinated five-hydration intermediates, $Mg(H_2O)_5^{2+}$.

	IP1	IP2	IP3	IP4	D1-SSH	D2-SSH	D3-SSH	D1-CP	D2-CIP	D3-CIP	D4-CIP
CH3COO			■			■				■	
HS			■			■				■	
HCO3		■			■					■	
CIT		■			■				■		
PHENAM		■			■				■		
C2O4		■			■				■		
SO4		■				■			■		
MAL		■				■			■		
GLU		■				■				■	
GLY		■				■				■	
SAL		■				■				■	
H2PO4		■				■		■			
ASP		■				■				■	
HCOO		■				■				■	
TAU		■				■				■	
F		■				■				■	
PEG	■				■						
IPA	■				■					■	
HPO4	■					■		■			
OH	■					■					
SIO3	■						■			■	
PHEN	■						■			■	
SIF6				■		■				■	
CLO4		■					■				■
Cl				■			■				■
I				■			■				■
NO3				■			■				■
PO4	■							■			
TAR						■			■		
